# Deciphering H3K4me3 broad domains associated with gene-regulatory networks and conserved epigenomic landscapes in the human brain

**DOI:** 10.1038/tp.2015.169

**Published:** 2015-11-17

**Authors:** A Dincer, D P Gavin, K Xu, B Zhang, J T Dudley, E E Schadt, S Akbarian

**Affiliations:** 1 Department of Psychiatry, Friedman Brain Institute, Icahn School of Medicine at Mount Sinai, New York, NY, USA; 2 Department of Genetics and Genomic Sciences, Institute for Genomics and Multiscale Biology, Icahn School of Medicine at Mount Sinai, New York, NY, USA; 3 Department of Neuroscience, Friedman Brain Institute, Icahn School of Medicine at Mount Sinai, New York, NY, USA; 4 Department of Psychiatry, Jesse Brown Veterans Affairs Medical Center, Chicago, IL, USA

## Abstract

Regulators of the histone H3-trimethyl lysine-4 (H3K4me3) mark are significantly associated with the genetic risk architecture of common neurodevelopmental disease, including schizophrenia and autism. Typical H3K4me3 is primarily localized in the form of sharp peaks, extending in neuronal chromatin on average only across 500–1500 base pairs mostly in close proximity to annotated transcription start sites. Here, through integrative computational analysis of epigenomic and transcriptomic data based on next-generation sequencing, we investigated H3K4me3 landscapes of sorted neuronal and non-neuronal nuclei in human postmortem, non-human primate and mouse prefrontal cortex (PFC), and blood. To explore whether H3K4me3 peak signals could also extend across much broader domains, we examined broadest domain cell-type-specific H3K4me3 peaks in an unbiased manner with an innovative approach on 41+12 ChIP-seq and RNA-seq data sets. In PFC neurons, broadest H3K4me3 distribution ranged from 3.9 to 12 kb, with extremely broad peaks (~10 kb or broader) related to synaptic function and GABAergic signaling *(DLX1*, *ELFN1*, *GAD1*, *IGSF9B* and *LINC00966*). Broadest neuronal peaks showed distinct motif signatures and were centrally positioned in prefrontal gene-regulatory Bayesian networks and sensitive to defective neurodevelopment. Approximately 120 of the broadest H3K4me3 peaks in human PFC neurons, including many genes related to glutamatergic and dopaminergic signaling, were fully conserved in chimpanzee, macaque and mouse cortical neurons. Exploration of spread and breadth of lysine methylation markings could provide novel insights into epigenetic mechanism involved in neuropsychiatric disease and neuronal genome evolution.

## Introduction

More than 100 amino-acid residue-specific histone post-translational modifications (PTMs) exist in the vertebrate cell.^1^ These PTM include mono (me1), di (me2)- and tri (me3) methylation, acetylation and crotonylation, polyADP-ribosylation and small protein (ubiquitin, small ubiquitin-like modifier) modification of specific lysine residues, as well as arginine (R) methylation and citrullination, serine (S) phosphorylation, tyrosine (T) hydroxylation and several others.^1, 2, 3^ Different combinations of the site- and residue-specific PTMs show differential enrichment across the genome, and some of the best-studied histone PTM are defined in the context of transcriptional regulation. For example, many active promoters show high levels of histone H3 lysine-4 methylation.^4^ In particular, the trimethylated form, H3-trimethyl lysine 4 (H3K4me3), with the lysine residue's side chain carrying three methyl groups, is primarily distributed in the form of sharp peaks, extending in neuronal chromatin on average only across 1000–1500 base pairs or less, with the large majority of peaks, or at least 70–80%, positioned within 2 kb of annotated transcription start sites (TSSs).^5^ However, in some tissues, a subset of sequences epigenetically decorated with H3K4me3 tend to stretch across several kilobases, with the broadest domains measuring up to 60 kb in length.^6, 7^ Recently, it was proposed that these broader H3K4me3 peaks show strong association with genes expressed in a cell-type-specific pattern, and could have an important role for transcriptional regulation by controlling RNA polymerase-II pausing as a critical variable for general elongation efficiency, and by reducing transcriptional noise.^7^ Furthermore, the finding that aspects of transcription including H3K4me3 breadth at TSS are linked to cell identity is extremely interesting. This is because until now, epigenetic signatures that critically distinguish between different cell types and tissues otherwise sharing the same genome were mostly confined to distant-acting *cis*-regulatory enhancer elements (p300, CBP, H3K4me1 and H3K27ac),^8, 9^ including exceptionally large enhancer domains called super-enhancers, which distinguish from the traditional enhancer concept by the very high occupancy of transcription apparatus such as the mediator complex and cell-type-specific transcription factors to drive expression of associated genes.^10, 11, 12, 13, 14^

However, currently very little is known about the regulation of the broadest H3K4me3 peaks in the human brain. This is surprising, given that regulators of H3K4 methylation significantly contribute to the genetic risk architecture of autism^15^ and to epigenomic alterations in autism and schizophrenia brain.^16, 17^ H3K4me3 landscapes in human cerebral cortex are subject to highly dynamic regulation throughout a broad window of development, extending deep into or even beyond childhood.^18^ The goal of the present study was to characterize the broadest H3K4me3 peaks from human prefrontal cortex (PFC), with comparative analyses in non-human primate and rodent, in the context of cell-type-specific regulation, association with neuronal and non-neuronal gene expression and potential implications for neurodevelopmental disease. Our study employs a broad range of bioinformatics approaches on next-generation sequencing-based transcriptomes and epigenomes from sorted neuronal and non-neuronal nuclei from PFC gray and white matter and, for comparison, peripheral blood mononuclear cells.

## Materials and methods

### Human subjects

All human subject work was approved by the Institutional Review Board of the University of Illinois at Chicago. Forty milliliter blood samples were collected from three male non-psychiatric control subjects (Supplementary Table 1), using a previous published protocol^19^ with minor modifications. Whole blood was diluted 1:1 with Hanks balanced salt solution (Invitrogen, Grand Island, NY, USA), then peripheral blood mononuclear cells were isolated using Ficoll-Paque Plus (GE Healthcare Lifesciences, Pittsburgh, PA, USA). Peripheral blood mononuclear cells, of which a large majority are lymphocytes, were then washed with Hanks balanced salt solution, and once more with phosphate-buffered saline. Samples were then flash frozen and stored at −80 °C until shipping on dry ice.

### Human postmortem brain samples and demographics

Human postmortem PFC brain tissue from 25 controls without known neurological or psychiatric disease (male=18, female=7, mean±s.d. age =30.8±29 years, postmortem interval = 14.75± 9 h, pH=6.5±0.3) were obtained from different brain banks including the University of Maryland Brain and Tissue Bank for Developmental Disorders, the Harvard Brain Tissue Resource Center, the Department of Psychiatry at the University of California at Irvine, and the Maryland Psychiatric Research Center in Baltimore (see Supplementary Table 1 for additional information for each specimen, including age of death, gender and postmortem interval and tissue pH). Each brain bank obtained the consent to use brain tissue for research from each individual or their guardian before his/her death, and their protocols were approved by their respective Institutional Review Boards. No individual-specific identifiable information was obtained by the investigators of this study.

### Animals

All animal experiments were approved by the Animal Use and Care Committee of the Icahn School of Medicine at Mount Sinai. Cerebral cortex from two adult wild-type mice was included in this study. Chromatin immunoprecipitation and next-generation sequencing (ChIP-seq) data sets from seven non-human primates (four chimpanzees and three macaques)^20^ were reanalyzed and included in this study (see Supplementary Table 1 for additional information for each specimen, including age of death, gender and postmortem interval). All non-human primate work was conducted on brain specimens collected after death.

### NeuN sorting via FACS and H3K4Me3 chromatin immunoprecipitation

Nuclei extraction was carried out as previously described.^5, 21^ Nuclei from freshly frozen (never fixed) tissues (250 mg per sample) of postmortem PFC from 25 healthy control subjects were extracted in hypotonic lysis buffer that causes the cells to swell to liberate nuclei. Nuclei were purified by ultracentrifugation and resuspended in 1x phosphate-buffered saline. Neuronal nuclei were immunotagged with anti-neuronal nucleus antibody (Millipore 377, Billerica, MA, USA) and sorted into NeuN(+) and NeuN(−) populations using a FACS Vantage SE flow cytometer. Chromatin was prepared by micrococcal nuclease (MNase) digestion of isolated neuronal nuclei (from minimum 1 × 10^6^ sorted nuclei), because MNase-treated chromatin is more efficient for subsequent immunoprecipitation with specific anti-methyl histone antibodies than crosslinked and sonicated chromatin in brain tissue. Purified mononucleosomal DNA were pulled downed using anti-H3K4me3 antibody (Upstate/Millipore 07473) with chromatin immunoprecipitation assay and then purified. ChIP-seq libraries were prepared from the immunoprecipitated DNA by blunt-ending, A-tailing and ligation to adaptors and PCR amplification for single-end sequencing (36-bp reads). All libraries were sequenced by an Illumina Genome Analyzer II (GA II, San Diego, CA, USA) platform.

### H3K4me3 chip-seq analysis pipeline

The ChIP-seq data analysis was performed using several bioinformatic tools and in-house python and perl scripts. Sequencing read quality was evaluated using FastQC (version 0.10.1 http://www.bioinformatics.bbsrc.ac.uk/projects/fastqc/). A low-quality read filter was then applied in which no reads with more than six bases with a minimum phred quality score of 20 were retained. Supplementary Table 2 reports summary alignment statistics for the total set of 41 ChIP-seq libraries (*n*=39 H3K4me3 ChIP-seq plus two input libraries (nucleosomal DNA prepared by micrococcal nuclease digest). Unique alignment percentages were between 63 and 96% of all total reads across all samples (Supplementary Table 2). Single-end 36-bp sequencing reads from the ChIP and input control libraries were aligned to the Human Reference Genome (National Center for Biotechnology Information build 37 (UCSC hg19)) using Bowtie (version 0.12.7) with parameters specified to report the best alignment allowing no more than one mismatch and excluding reads that aligned to more than one location in the genome. H3K4me3 broad peaks were identified using MACS version 2.0.10.20131216 (tag:beta)^22^ with —broad-cutoff=0.1 —mfold=10,30 —qvalue=0.01 parameters on pooled data. Reads were pooled across cell-type-specific samples, cohort 1 NeuN+ (*n*=11), cohort 2 NeuN+ (*n*=14), NeuN− (*n*=2), blood (*n*=3), for each cell type separately. Peaks were annotated to genes and TSSs using HOMER annotatePeaks.pl script with UCSC refGene.^23^ All aligned read files were corrected for sequencing depth using the signal extraction method proposed by Diaz *et al.*^24^ and normalized to the cell-type-specific input to visualize in Integrative Genome Viewer browser.^25^ For Gene Ontology (GO) term analyses, we used two approaches: gene- and peak-based coordinates by using the web interface of DAVID, Stanford's Genomic Regions Enrichment of Annotations Tool^26^ and the R package ChIPEnrich (http://sartorlab.ccmb.med.umich.edu/chip-enrich). Additional annotation and analysis including general assessments of overlaps between bed-files and to extract signal intensity scores for defined regions was performed using BEDTools v2.17,^27^ Pybedtools,^28^ SAMTools,^29^ UCSC tools,^30^ BSGenome, GenomicFeatures, rtracklayer and ChIPpeakAnno packages in R (http://www.bioconductor.org). The snapshots of the H3K4me3 epigenomic profiles were obtained with Integrative Genome Viewer browser.^25^ Heatmaps displaying normalized read densities of ChIP-seq samples were generated with the deepTools package.^31^ This ChIP-seq pipeline was applied to the other species, by aligning reads for the chimpanzee to the panTro4, the macaque monkey to rheMac2 and the mouse to the mm9 genome, respectively.

### RNA-seq

RNA was extracted from ∼75 mg of gray and white matter dissected from seven adult control PFC specimens (Supplementary Table 1) using the RNeasy Lipid Tissue Mini kit (catalog #74804, Qiagen, Hilden, Germany), treated with DNase I, purified and diluted to 20 ng μl^−1^. Sequencing libraries were prepared according to the NuGen Ovation RNASeq version 2 protocol, and run on the paired-end 50-bp module in Illumina HiSeq 2000 (Eurofins MWG; Operon). RNA-sequencing (RNA-seq) raw reads that passed the quality control metric, which is referred to as the ‘chastity filter' by Illumina, were aligned to the UCSC *Homo sapiens* reference genome build 19 using the STAR aligner^32^ and were visualized using the Integrative Genome Viewer.^25^ A raw read-count table was generated using HTSeq python framework.^33^ Alignment percentages were between 74 and 86% of all total reads across all samples (Supplementary Table 2). Expression of genes and transcripts of the resulting aligned bam files were quantified with Cufflinks v1.3.0, which assembles transcripts and estimates their abundances in RNA-seq samples^34^ by using UCSC gene annotation file (hg19 in GTF format) as a guiding gene model set. After all short read sequences were assembled into transcripts, their relative expression levels were measured in fragments per kilobase of exon per million fragments mapped (FPKM) unit, where read counts are normalized by the transcript length (exon only) as well as the total number of mappable reads in the sequencing library. Box plot of FPKM distributions for each gene was plotted with the R package ggplots2. To validate neuronal (NeuN+)-specific and non-neuronal (NeuN−)-specific broadest H3K4me3 peaks within the 4-kb window of TSSs of the associated genes, we further investigated differentially expressed genes quantified by DESeq2, limma-voom and edgeR, in subcortical white (*n*=6) and cortical gray matter (*n*=6).

### Functional enrichment

We employed the Genomic Association Test^35^ to examine whether the broadest neuron-specific H3K4me3 genomic intervals are associated with functional elements more than expected by chance via simulation within a genomic context including corrections for gene density, chromosomal segments and isochore structure to prevent confounding effects due to different G+C content and provide unbiased measures of the null expectation.^30^ Before the association analysis, a custom perl script was used to query the public instance of the UCSC MySQL database of the human genome version hg19 at the host genome-mysql.cse.ucsc.edu and created a set of non-overlapping intervals of RefSeq transcripts covering the full genome including promoter, intergenic, intron, CpGislands Centromeres, microRNA, noncoding RNA and exonic regions. Using the bioinformatic analysis tool ROSE Rank Order of Super-Enhancers software (http://younglab.wi.mit.edu/super_enhancer_code.html), 357 super-enhancers and 5120 typical enhancer loci were ranked from H3K27Ac ChIP-seq data of the brain middle frontal lobe. Other brain-related genomic intervals were downloaded from Fantom5 (refs 36, 37) nd published data sets on PFC neurons.^16, 18^ We computed an expected count using 10 000 randomized simulations of the 523 top 5% broadest neuron-specific H3K4me3-binding intervals taking into account the observed segment length distribution. Supplementary Table 3 shows the fold enrichment as the ratio of observed and expected overlap with an empirical *P*-value for associations of the respective brain genomic features with 523 top 5% broadest neuron-specific H3K4me3-binding intervals. Overlap of genomic intervals was assessed using BEDTools.^27^

### *De novo* motif analysis and comparison with known motifs

For motif analyses, including enrichment and comparative matching of *de novo* sequences, five databases were combined, Transfac,^38^ Jaspar,^39^ Uniprobe,^40^ hPDI^41^ and Taipale.^42^ *De novo* motifs were compared against a total of 3764 motifs by EPIGRAM^43^ using k-mer features with lasso regularization to characterize motifs associated with H3K4me3 in NeuN+ nuclei. TOMTOM (version 4.9.1) tool from the MEME suite was used to identify nearest matches of the discovered *de novo* motifs identified from EPIGRAM against a total of 3764 motifs.

### Construction of PFC weighted gene co-expression network

The absolute Pearson correlation matrix S=[*S*_ij_] for all possible pairwise genes were converted into an adjacency matrix A=[*a*_ij_] by power function, that is, *a*_ij_ *power*(*S*_ij_, β)≡|*S*_ij_|^β^. The value of the power adjacency function's exponent (*β*) was chosen using the scale-free topology criterion proposed in the study by Zhang and Horvath^44^ to ensure the resulting weighted network exhibited an approximate scale-free topology and a high mean number of connections. To explore the modules of the co-expression network, the adjacency matrix was further transformed into a topological overlap matrix to filter very weak connections and to provide more cohesive and biologically meaningful modules. Topological overlap matrix-based dissimilarity measure between all possible pairwise genes was used as input in average linkage hierarchical clustering, followed by a Dynamic Tree cut algorithm to define modules as branches of the resulting cluster tree.^45^ Each module was assigned a unique color identifier and gray color representing poorly connected genes. Highly co-expressed genes have a small dissimilarity. With the biologically motivated data reduction scheme, we wanted to explore and identify modules of highly co-regulated genes from the 475 broadest H3k4me3 domains within the 4-kb window of refseq genes.

### Construction of Bayesian gene networks

A Bayesian network is used to construct gene networks based on a previously described data set of gene expression profiles from 173 PFC samples from non-demented healthy individuals using a Bayesian network reconstruction algorithm implemented in the RIMBANET package.^46, 47, 48^ The resulting PFC control network was visualized by Cytoscape 3.1.1 (ref. 49) and integrated with 475 NeuN+-specific peaks that annotated to the ±4 kb-window of TSS of respective genes. Network Node statistics are computed by treating as undirected network using Cytoscape 3.1.1.

### Accession

ChIP-seq and RNA-seq data sets newly generated for this manuscript are deposited in the National Center for Biotechnology Information, accession no. GSE71238. For previously published data sets, see accession no. GSE21172 and additional links provided in the study by Shulha *et al.*^16,18,20^ (for: Shulha *et al.*^16, 18^ https://zlab.umassmed.edu/zlab/publications/ShulhaPLOSGen2013.html; https://zlab.umassmed.edu/zlab/publications/ShulhaAGP2011.html). For Shulha *et al.*,^20^ sequences are accessible through http://www.umassmed.edu/zlab/publications/).

## Results

We isolated nuclei from the rostral PFC for separation and fluorescence-activated sorting (FACS) based on immunolabeling with the NeuN antibody (which binds to the overwhelming majority of neuronal nuclei in the cerebral cortex), followed by ChIP-seq for genome-scale mapping of the H3K4me3 mark in neuronal and non-neuronal chromatin.^5^ Altogether, our study was comprised of H3K4me3 ChIP-seq data sets from 30 subjects, including 25 NeuN+ and 2 NeuN− samples from the PFC of 25 postmortem brains, and three additional samples of peripheral blood mononuclear cells obtained by venipuncture from living subjects. Two of the NeuN+ samples and all blood-derived samples were newly generated for this study, whereas the remaining samples from brain nuclei had been included in previous publications on cell-type-specific^5^ and developmental regulation^18^ of H3K4me3 peaks in the PFC.

We first examined cell-type-specific regulation of the broadest H3K4me3 peaks (top 5% broadest H3K4me3 peaks (length in base pairs) that were longer than the 95th percent of all H3K4me3) in a cohort of 11 PFC NeuN+ samples (‘cohort 1' in Supplementary Table 1). To characterize the cell-type specificity of these top 5% broadest methylation peaks, we compared the data set from cohort 1 with the two non-neuronal samples from the PFC, and with the three blood samples. We identified H3K4me3 peaks that were specific to each cell type in our data set and then verified the NeuN+ specific peaks in an independent set of 14 additional NeuN+ PFC samples (‘cohort 2' in Supplementary Table 1).

**Figure 1:**
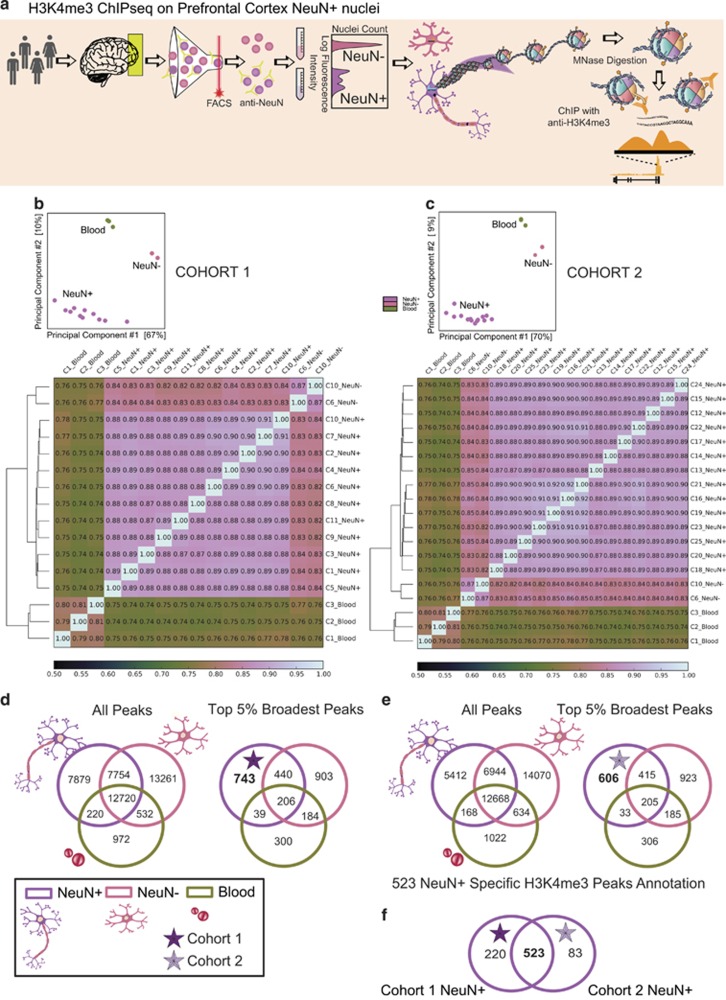
Cell-type-specific histone methylation profiling in PFC. (**a**) Graphical outline of experiment starting with postmortem cerebral cortex (PFC) to generate cell-type-specific H3K4me3 maps. (**b**, **c**) Heatmaps for Spearman's rank correlation coefficients comparing H3K4me3 profiles for three peripheral mononuclear blood cells (blood), two sorted NeuN− PFC cells, compared with (**b**) 11 PFC NeuN+ samples (cohort 1) and (**c**) 14 PFC NeuN+ samples (cohort 2) each from a different individual, showing much higher correlations between samples from the same cell type as compared with sample correlations across cell types and tissues. Principal component analyses showing complete separation of NeuN+ samples from other cell types with the first two principal components. (**d**, **e**) Venn diagrams showing absolute number of peaks for NeuN+, NeuN− and blood, confirming for (**d**) cohort 1 and (**e**) cohort 2 the enrichment cell-type-specific peaks among the top 5% broadest H3K4me3 peaks, as compared with the total set of peaks. (**f**) Venn diagram confirming that large majority of neuron-specific peaks from cohort 1 are confirmed in replication sample (cohort 2). ChIP-seq, chromatin immunoprecipitation and next-generation sequencing; H3K4me3, H3-trimethyl lysine 4; PFC, prefrontal cortex.

As a quality control and validation of ChIP-seq experiments, we compiled the H3K4me3 epigenomic landscape patterns for RefSeq-annotated genes across all samples and computed the pairwise Spearman's rank correlation coefficients as a measure of similarity. The Spearman's rank correlation coefficients between PFC neuronal samples (ranging from 0.88 to 0.92) were consistently higher, compared with the correlations observed between neuronal and non-neuronal PFC cells (ranging from 0.82 to 0.87) or blood (0.74–0.76). In fact, across these different cell types, the intra-cell-type correlations were systematically higher than inter-cell-type correlations, each cell type clustering together in an unsupervised pattern (Figures 1a–c). Although the Spearman correlation heatmaps showed clustering based on read coverage at REFSEQ genes, we performed a principal components analysis on the fingerprints of the samples based on peak loci identified by MACS, in which a binding affinity matrix containing a normalized read count for each subject at every potential H3K4me3-binding site, comparing PFC NeuN+ neurons from cohorts 1 and 2 separately, with the non-neuronal PFC samples and blood (Figures 1b and c). Strikingly, for both cohort 1 and 2, all neuronal samples, ranging in age from 0.5 to 81 years, are located at one end of the graph, far from the space occupied by the non-neuronal NeuN- samples from the same tissue (PFC) and from blood (Figures 1b and c). For two subjects, both NeuN+ and NeuN− PFC nuclei were examined by ChIP-seq. Nonetheless, H3K4me3 landscapes in the NeuN+ samples from these two subjects were much more similar to the profiles of other NeuN+ samples, as compared with the non-neuronal (NeuN−) nuclei that had been extracted in parallel (to the NeuN+ sample) from the same PFC donor tissue (Figures 1b and c). Therefore, our Spearman correlation analyses reveals that H3K4me3 in brain cells is heavily regulated in a cell-type-specific manner, which on a genome-wide scale is much more prominent than any subject-specific signatures.

### Cell-type-specific enrichment for broadest H3K4me3 peaks

H3K4me3 breadth had been implicated in cell identity in various cell lines in a recent study.^7^ Therefore, we wanted to explore whether the broadest H3K4me3 peaks in our data sets show numerical evidence for cell-type-specific regulation. To this end, we first calculated the average H3K4me3 peak length in our NeuN+ sample, which was ~1.5 kb both in cohort 1 and cohort 2 across all 28573 H3K4me3 peaks and 25212 H3K4me3 peaks identified, respectively (Supplementary Figure 1C). We then selected the broadest 5% of H3K4me3 peaks, which on average extended across 5.2 kb (cohort 1=1428 top 5% broadest H3K4me3 peaks) and 5.4 kb (cohort 2=1259 top 5% broadest H3K4me3 peaks). The absolute range of the top 5% peaks was 3.9–10 kb (Supplementary Figure 1C). There were a total of 523 H3K4me3 peaks consistently found among the top 5% widest peaks specifically present in all 25 PFC NeuN+ samples of cohorts 1 and cohort 2. These 523 peaks represented the overlap between the top 5% (broadest) peaks of cohort 1 (*n*=743 peaks) and top 5% (broadest) peaks of cohort 2 (*n*=606 peaks), indicating that a large majority of broad, neuron-specific H3K4me3 peaks are highly reproducible (Figures 1d–f; Supplementary Table 4). In order to test whether two sets of intervals of NeuN+-specific peaks from cohort 1 and cohort 2 are related spatially, we randomly shuffled the genome and checked the observed versus simulated (shuffled) regions to calculate the significance of overlaps by performing 1000 random permutations. This overlap between cohort 1 and cohort 2 was highly significant (*P*<0.001) (permutation test, based on 1000 random shufflings of H3K4me3 peaks genome-wide). Similarly, nucleated blood cells and non-neuronal PFC cells harbored 297 (blood) and 867 (NeuN− in PFC) cell-type-specific peaks among the top 5% broadest H3K4me3 peaks (Supplementary Tables 5 and 6). The broadest (top 5%) H3K4me3 peaks, as a group, were 1.9- and 2.2-fold more likely to be cell-type specific, compared with the total set of H3K4me3 peaks (Figures 1d–f). These enrichments were highly significant (Fisher's exact test, *P*<2.2e−16; cohort 1: broadest 5% NeuN+ specific/total set of peaks, *n*=743/28 573; all NeuN+ specific/total set of peaks, *n*=7879/28 573; cohort 2: broadest 5% NeuN+ specific/total set of peaks, *n*= 606/25 212; total NeuN+ specific/non-specific peaks, *n*= 5412/25 212) (Figures 1e and f). Previous studies^7^ used the 5% threshold to characterize the cell-type-specific nature of the broadest H3K4me3 peaks. We examined more stringent cutoffs and this resulted in even stronger effects by cell type. For example, top 1% broadest peak cell-type-specific enrichments were cohort 1; 2.4-fold (top 1%) versus 1.9-fold (top 5%) and cohort 2, 2.9-fold (top 1%) versus 2.2-fold (top 5%) (Supplementary Figures 1A and B). We conclude that the majority of ‘extremely stretched' H3K4me3 peaks in neurons are subject to cell-type-specific regulation.

**Figure 2:**
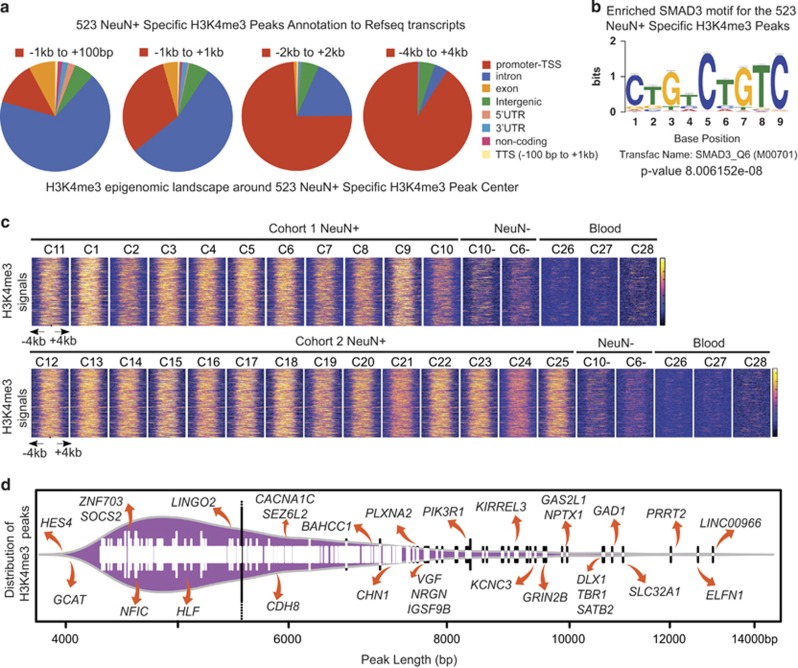
Features associated with top 5% broadest NeuN+ neuron-specific H3K4me3 peaks. (**a**) Pie charts express proportions of 523 (top 5%) broadest NeuN+-specific H3K4me3 peaks in relation to Refseq database, including ‘promoter TSS', intron, exon, intergenic, 5′UTR, 3′UTR and TTSs, as indicated. Pie charts are defined from left to right by increasing the window size of ‘promoter TSS'. Note that overwhelming proportion of peaks (>85%) are found within 4 kb of annotated Refseq TSSs. (**b**) Top sequence motif enriched in broadest domain (top 5%) neuronal H3K4me3 peaks. (**c**) Clustered H3K4me3 profiles within a ±4-kb window for the 523 (top 5%) broadest NeuN+-specific H3K4me3 peaks. The strength of the H3K4me3 signal is shown on a color scale of yellow (strong) to red (intermediate) to blue (weak) signal. Notice highly consistent enrichments across all subjects' PFC NeuN+ samples in cohorts 1 and 2, in comparison with non-neuronal (NeuN−) cells and blood. (**d**) Bean plot showing distribution of 523 NeuN+ broad peaks ordered by breadth of H3K4me3 signal, with broadest peak (*LINC00966*) extending across ~13 kb. Purple area, estimated density of peak distribution. Tall vertical line, average bp length of top 5% broadest peaks (~5.4 kb). H3K4me3, H3-trimethyl lysine 4; TTS, transcription termination site; UTR, untranslated region.

In neurons, the overwhelming majority of broad H3K4me3 peaks were located within 4 kb of an annotated TSS in the REFseq database (Figure 2a). Furthermore, at least 13 of the broad H3K4me3 peaks called for ‘intergenic' sequences (which comprised ~ 5% of the total pool of 523 broadest peaks) matched to non-annotated (novel) transcripts in our RNAseq data sets (for example: chr4: 565745-573996) (Supplementary Table 7). Therefore, the broadest H3K4me3 peaks, as a group, are primarily associated with the 5′ end of gene transcripts, with ~ 85% of broad peaks within 4 kb from the nearest TSS (Figure 2a). Furthermore, motif analysis, based on five independent databases (see Materials and Methods section), revealed highest enrichment (*P*<10^−7^) for SMAD3, a member of the SMAD family of transcription factors, and additional transcription factors with weaker enrichments (Figure 2b; Supplementary Table 8). These motifs were specific for the top 5% broadest neuronal peaks, whereas the total pool of neuronal H3K4me3 peaks was defined by differential enrichment of the ‘housekeeping' transcription factor SP1. Furthermore, the length of the 523 (top 5%) broadest H3K4me3 neuron-specific peaks was highly consistent across each NeuN+ sample from cohort 1 and cohort 2, and in addition, showed consistently only very weak signals in non-neuronal PFC cells and in blood (Figure 2c). Many genes with a prominent role in neuropsychiatric disease were found among the group of broadest peaks (Figure 2d; Supplementary Table 4). For example, extremely broad (>9 kb in length) H3K4me3 peaks were found at *KCNC3*, encoding a voltage-gated potassium channel linked to spinocerebellar ataxia, a neurodegenerative condition,^50^ and at the site of multiple neurodevelopmental risk genes including NMDA glutamate receptor subunit *GRIN2B* and transcription factor *SATB2* (refs 51, 52, 53) (Figure 2d). The two broadest H3K4me3 peaks in PFC neurons, extending >12 kb, were *ELFN1* (extracellular leucine rich repeat fibronectin domain 1), implicated in epilepsy and attention-deficit hyperactivity disorder and essential for GABAergic signaling in subsets of cortical and hippocampal interneurons^54, 55^ and *LINC00966*, a poorly characterized noncoding transcript that encodes within its sequence microRNA 124-2, targeting homeobox transcription factor *Dlx5* (ref. 56) with a critical role in cortical interneuron development^57^ (Figure 2d). Of note, the group of extremely broad peaks includes additional key genes for GABAergic circuitry, including *DLX1* and *GAD1* (refs 58, 59) and *IGSF9B*^60^ (Figure 2d). This strong enrichment and overrepresentation of neuronal genes was very specific for the top 5% broadest NeuN+ H3K4me3 peaks. For example, we calculated the tallest NeuN+ peaks for each cohort and identified reproducible top 5% tallest NeuN+ peaks in cohorts 1 and 2 (Supplementary Tables 9 and 10). However, for this collection of top 5% tallest peaks (*n*=151 reproducible in cohorts 1 and 2), enrichments (incl. cell type and function) were overall very weak and modest, with no evidence for biological enrichment (Supplementary Table 11).

**Figure 3:**
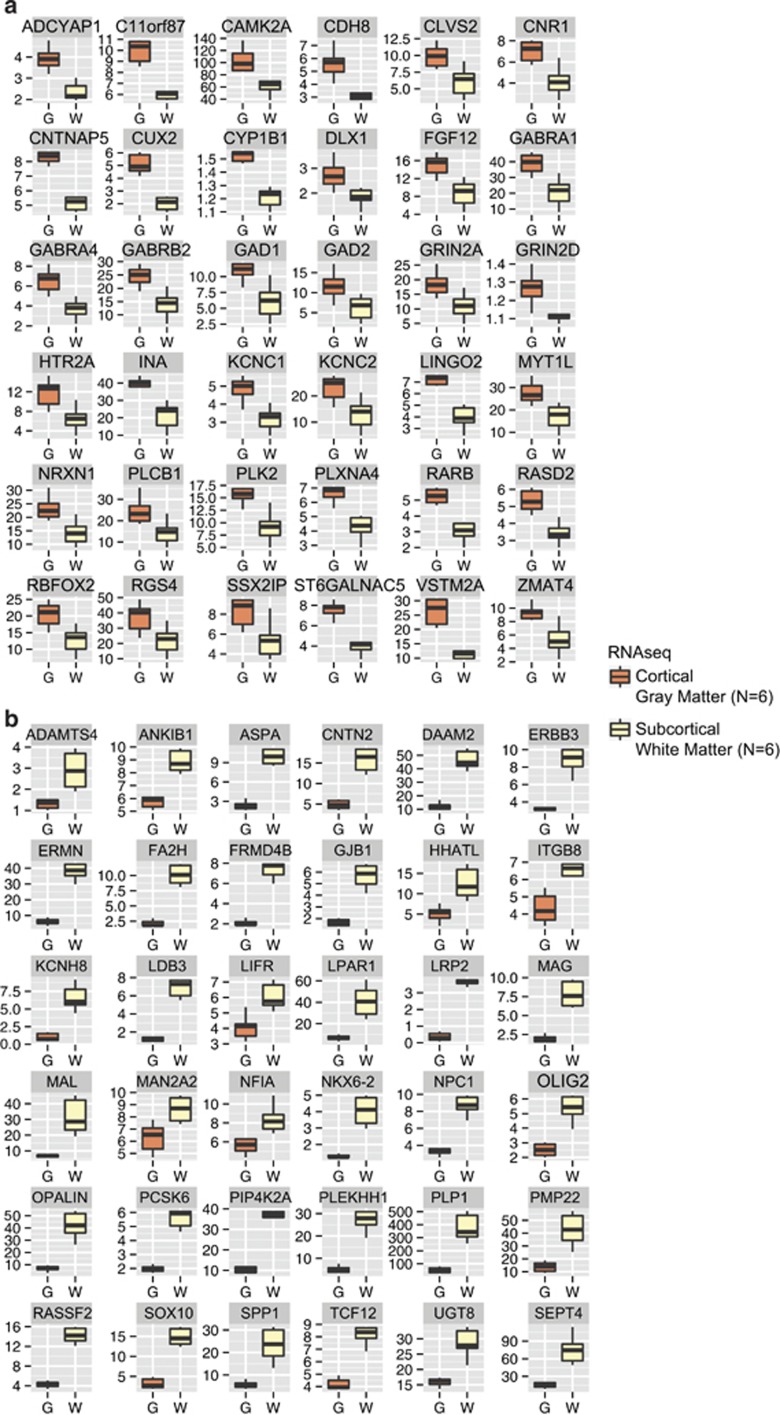
Transcripts associated with top 5% broadest NeuN+ and NeuN−-specific H3K4me3 peaks. (**a**) Box plots comparing for 36 representative gene transcripts the gray (G) and white matter (W) RNA-seq signal from PFC tissue blocks of six subjects, showing much higher FKPM/expression in G compared with W. All transcripts are within 4 kb of a top 5% broadest H3K4me3 peak specific to PFC neurons. (**b**) Box plot comparison of PFC gray (G) and (W) white matter expression for 25 representative transcripts associated with broad non-neuronal H3K4me3 peaks (*n*=6 (G) gray and *n*=6 (W) white matter samples). FKPM, fragments per kilobase per million; H3K4me3, H3-trimethyl lysine 4; PFC, prefrontal cortex.

In peripheral tissues, the broadest (top 5%) H3K4me3 peaks reportedly are linked to cell-type-specific expression and ‘transcriptional consistency' contributing to steady-state RNA production.^7^ Therefore, we hypothesized that RNA transcripts associated with promoters from the top 5% broadest H3K4me3 peaks specific to neurons are more likely to show higher levels of expression in PFC gray matter as compared with the adjacent subcortical white matter. This is because neuronal densities are up to 150-fold higher in gray versus subcortical white matter.^61^ Therefore, we hypothesized that RNA-seq from neuron-enriched compartment (gray) will show much more robust expression for many of the NeuN+ (neuron specific) H3K4me3 peaks, as compared with RNA-seq from the neuron-depleted (white matter) compartment. To this end, we first identified 475 (from total *n*=523 broadest top 5%) peaks that overlapped (±4 kb) with the TSS of an annotated REFseq gene, then quantified for six postmortem PFC specimens by RNA-seq the corresponding transcripts separately for the PFC gray and for the underlying white matter dissected from same tissue blocks. Indeed, the majority of the 475 transcripts associated with broad H3K4me3 peaks in neurons expressed at much higher levels in PFC gray matter with its neuron-rich six cortical layers, compared with the (neuron depleted) subcortical white matter (*P*<0.0001, Wilcoxon matched-pairs signed rank test). These include multiple risk genes associated with neuropsychiatric disease such as multiple genes encoding various ion channels associated with synaptic signaling (*KCNC1*, *KCNC2*, *5HTR2A* and others, Figure 3a). In addition, we noticed that 427 transcripts positioned within 4 kb of top 5% broadest human NeuN+ H3K4me3 peaks matched transcripts in a transcriptome database for mouse cortical neurons and six glial and endothelial cell types.^62^ Indeed, expression for the large majority of the 427 transcripts was much higher in cortical neurons as compared with glia and endothelium (Supplementary Figure 2), a finding that provides further support for the robust association between top 5% broadest H3K4me3 peaks and cell-type-specific regulation.

Having shown that many of the broadest neuron-specific H3K4me3 peaks are associated with transcripts expressed at much higher levels in the six-layered PFC gray matter as compared with the underlying white matter (Figure 3a), we next wanted to explore whether, conversely, RNA transcripts associated with promoters from the top 5% broadest H3K4me3 peaks specific to non-neuronal (NeuN−) PFC cells are more likely to show higher levels of expression in PFC white matter as compared with the overlying gray matter. Indeed, the majority of 759 transcripts associated with the 867 (top 5%) broadest peaks specific for non-neuronal (NeuN−) PFC chromatin showed higher expression in PFC white as compared with gray matter (****P-value<0.0001, Wilcoxon matched-pairs signed rank test). These included *OPALIN*, *SEPT4*, *SOX10* and other key regulators for myelination and oligodendrocyte differentiation and function (Figure 3b).

### Functional annotations and conservation of the broadest neuron-specific H3K4me3 peaks

**Figure 4:**
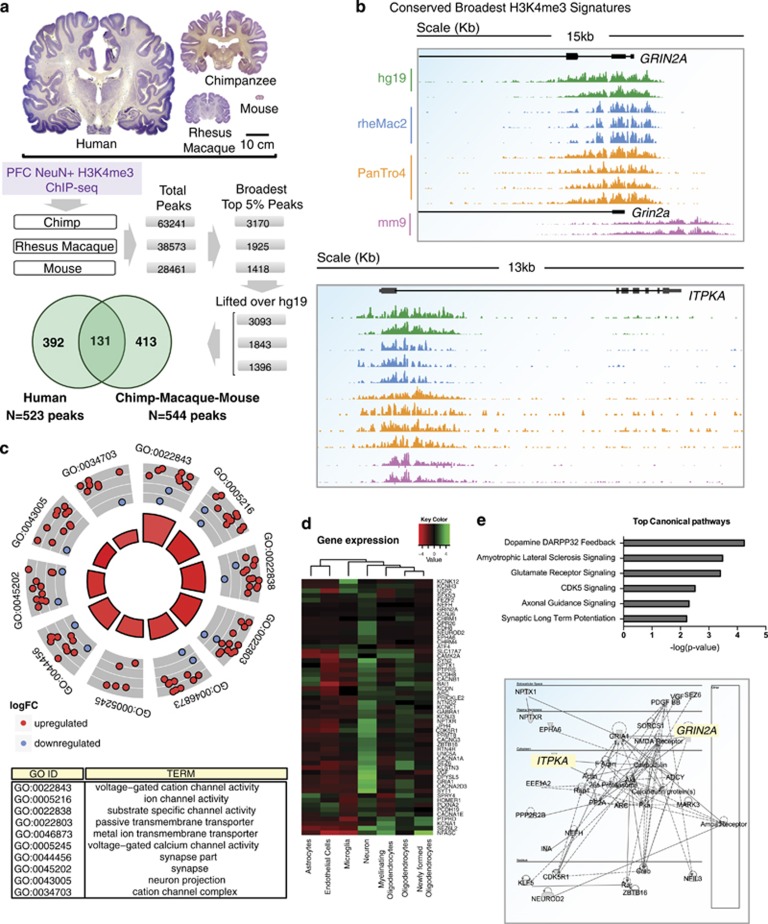
Conserved broadest H3K4me3 domains are associated with synaptic signaling and neuronal functions. (**a**) Number of H3K4me3 peaks, total and top 5% broadest in prefrontal neurons of chimpanzee, macaque and adult mouse cerebral cortex, before and after liftover into HG19, as indicated. Brain coronal sections reproduced with permission from http://www.brains.rad.msu.edu, and http://brainmuseum.org, supported by the US National Science Foundation. Venn diagram shows that 131/525 top 5% broadest human NeuN+ H3K4me3 peaks are conserved across the four species. (**b**) Representative examples for conserved broadest peaks. (**c**) Circosplot highlighting top 10 DAVID Gene Ontology categories, comprised of 55 genes (shown in **d**) with FDR (Benjamin Hochberg) *P*<0.05. Notice 10/10 are directly related to synaptic signaling. Red/blue dots represent log-fold change in expression (neurons vs non-neuronal cell type (oligodendrocyte) using the cell-type-specific transcriptome database,^62^ as indicated. (**d**) Transcriptome heatmaps from mouse cerebral cortex, comparing expression levels of 55 genes from 10 DAVID categories (**c**) across seven cell types, notice highest expression in cortical neurons for large majority of conserved broadest peak-associated transcripts. (**e**) (Top) Top 5 Ingenuity pathways significantly enriched in a pool of 131 conserved broadest neuronal H3K4me3 peaks. (Bottom) representative pathway example (behavior, cell-to-cell signaling), top 5% broadest peak-associated pathway genes highlighted in yellow. FDR, false discovery rate; H3K4me3, H3-trimethyl lysine 4.

**Figure 5:**
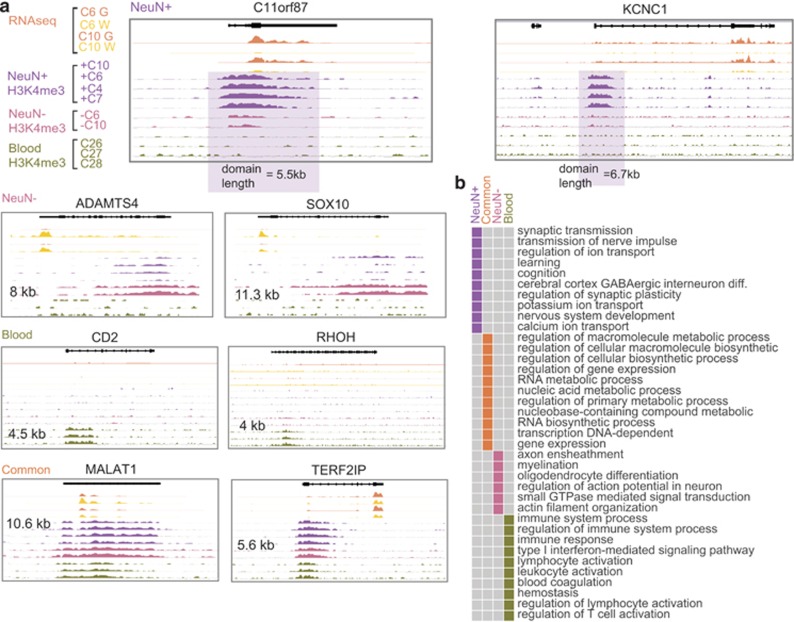
Cell-type-specific transcriptomics and epigenomics. (**a**) Browser tracks for eight genes subject to cell-type expression, showing (top to bottom) RNAseq tracks (orange/yellow) for PFC gray (G) and white matter (W) from subjects C6 and C10, as indicated. H3K4me3 ChIP-seq tracks from PFC NeuN+ (purple) and NeuN- (pink) nuclei of subjects and blood (green), from additional subjects. Note that robust expression in PFC gray matter (G) corresponds to strong H3K4me3 peaks in neuronal (NeuN+) chromatin. H3K4me3 domain length for each gene is listed in kb. (**b**) Lower-right panel shows examples of GO categories enriched in specific cell types (FDR-adjusted *P*<0.05, see the Results section), with synapse- and neuron-specific enrichments in PFC NeuN+ neurons (purple), myelination and oligodendrocyte footprint in PFC NeuN− cells (pink), immune functions in blood cells (green) and broadly defined metabolic pathways common to all three cell types (orange). ChIP-seq, chromatin immunoprecipitation and next-generation sequencing; FDR, false discovery rate; H3K4me3, H3-trimethyl lysine 4; PFC, prefrontal cortex.

Using the Genomic Regions Enrichment of Annotations Tool^26^ with multiple pathways (GO Mouse Phenotype, PANTHER and Pathway commons), we discovered for the 523 broadest top 5% neuronal peaks a strong footprint for neuronal function. GO terms enriched by binomial test for peak regions and hypergeometric test for mapped genes with an false discovery rate <0.05 were considered significant (Figure 4). For example, as it pertains to the top GO biological process, molecular function and cellular component categories, all were related to neuronal connectivity, development and synaptic plasticity and learning (Figure 5; Supplementary Table 12A). In contrast, the top three most specific GO biological process categories for the broadest top 5% H3K4me3 peaks from non-neuronal (NeuN−) PFC cells were axon ensheathment, myelination and oligodendroycte differentiation, whereas the broadest peaks in blood cells were enriched for immune system-related categories (Figure 5; Supplementary Table 12B). These findings, taken together, further affirm that the broadest H3K4me3 peaks show strong, cell-type-specific regulation in PFC neurons, with the majority of neuron-specific peaks depleted or absent in non-neuronal PFC cells and blood.

Comparative epigenomic studies across different primate species, reported species-specific regulation for a subset of H3K4me3 peaks in PFC neurons^20, 63^ and blood^64^ and furthermore, the PR/SET domain containing H3K4-specific methyltransferase *Prdm9*, regulating H3K4me3 in germline tissue, is thought to drive speciation in multiple mammalian lineages, including primates^65, 66^ and rodents.^67^ Therefore, we wanted to explore cross-species conservation of the top 5% broadest H3K4me3 peaks in cortical neurons, by comparing the PFC NeuN+ H3K4me3 ChIP-seq libraries from our human cohort with PFC NeuN+ H3K4me3 ChIP-seq libraries from four chimpanzees and three macaques, and cerebral cortex H3K4me3 ChIP-seq libraries from two adult C57Bl6 mice^68^ (Supplementary Tables 1 and 2). Of note, for each of the four species, the top 5% broadest H3K4me3 peaks were ~3.6-fold broader when compared with all H3K4me3 peaks called for that species (Supplementary Figure 3). Interestingly, in the human samples, overall H3K4me3 peak length appears to be shifted to include longer sequence stretches/peak (Supplementary Figure 3). These findings, taken together, would imply that the average length of the top 5% broadest H3K4me3 peaks, in relation to the overall population of H3K4me3 peaks, is constrained across species.

Next, for each animal sample, peaks (including broadest top 5%) called for that species' reference genome (macaque, *MMul1.0*; chimpanzee, *PanTro4*; mouse, *mm9*) were lifted over to the human reference genome *HG19* (Supplementary Figure 2). Interestingly, mouse, macaque and chimpanzee cortical neurons shared 544 of their top broadest 5% of peaks of which 131, or 24%, matched the top 5% broadest peak in human neurons (Supplementary Table 13). We performed pathway and upstream regulator analyses using QIAGEN's Ingenuity Pathway Analysis (IPA, QIAGEN Redwood City, www.qiagen.com/ingenuity) tools to detect molecular pathways that are enriched among these loci. Our analyses revealed dopamine-cAMP and glutamate receptor signaling were the most affected canonical pathways (Supplementary Tables 14 and 15).^69^ Similarly, the top 25 pathways with strongest *P*-values were overwhelmingly related to neuronal plasticity and signaling (Supplementary Table 16). We conclude that some of the broadest H3K4me3 domains show a high degree of epigenetic conservation across different mammalian lineages, including many genes regulating excitatory neurotransmission and monoaminergic pathways as critical modulators of attention, motivation and cognition.

### Broadest neuron-specific H3K4me3 peak-associated genes harbor special topological positions in a Bayesian and weighted gene co-expression network of human PFC

**Figure 6:**
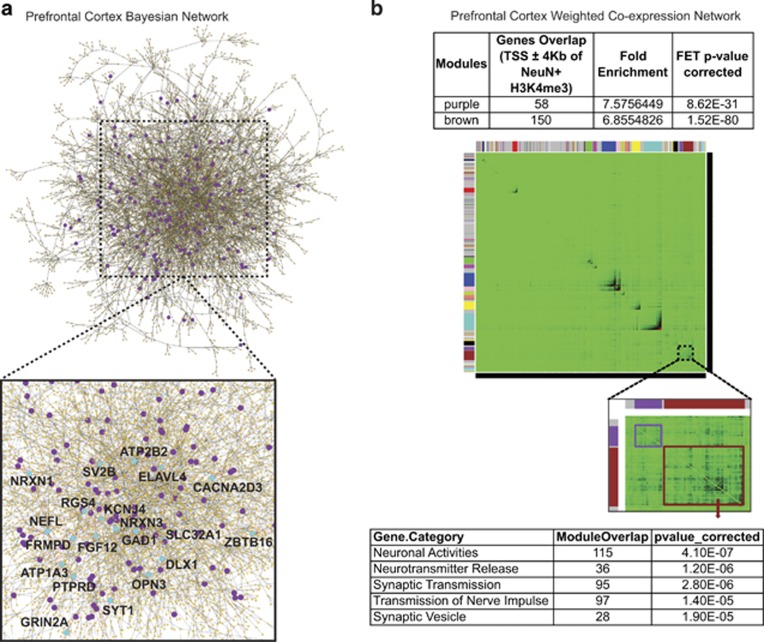
Integrative network-based analyses identify and prioritize key drivers associated with top 5% broadest neuronal H3K4me3 peaks. (**a**) Mapping 475 genes associated with top 5% broadest neuronal H3K4me3 peaks in the constructed Bayesian network of human prefrontal cortex revealed that only 158 genes (out of 475 genes) fell in the center of the network composed of about 7000 genes and 8000 edges. On the basis of the figure, there is not strong concentration in the center of the network; however, statistics show that the 158 neuron-specific genes do have significant higher degree and significant smaller average path lengths than the other genes in the network. Network statistics comparison suggesting that these genes actually tend to be located in the middle of the network (Supplementary Tables 17). The purple nodes are representing genes that annotated to 4 kb TSS −4 kb. Blue nodes representing a subset of genes, including the synaptic vesicle gene *SV2B* and the NMDA receptor subunit *GRIN2A* were among the top three genes in multiple network categories (see Supplementary Figure 18; *SV2B* in ‘average shortest pathway', *SV2B* and *GRIN2A* in ‘between centrality', *SV2B* in ‘degree' and *SV2B* and *GRIN2A* in ‘stress'. (**b**) TOM plots of a weighted gene co-expression network constructed from PFC of normal control subjects (see the Materials and Methods section). In this symmetric TOM heatmap, the rows and the columns represent genes and color intensity (green: no connection and blue: strong connection) represents network connection strength between any pair of nodes (genes) based on topological overlap between genes. The brown and purple modules, highlighted with a brown and a purple box, respectively, overlap most significantly with the H3K4me3 peaks genes and are enriched for the neuronal activities and synaptic transmission signature. H3K4me3, H3-trimethyl lysine 4; PFC, prefrontal cortex; TOM, topological overlap matrix; TSS, transcription start site.

As genes can interact with each other in a non-linear fashion, networks are constructed to examine such interactions in a systematic way. Here we constructed a Bayesian network based on gene expression profiles from PFC samples from non-demented individuals (see the Materials and Methods section). We focus on the largest connected component, which includes 7040 genes. In all,158 out of the 475 broadest neuron-specific H3K4me3 peak-associated genes (defined as annotated TSS within 4 kb from a top 5% broadest peak) are found to be within this component of the network (Figure 6a; Supplementary Table 17). These 158 broadest peak-associated network genes included key regulators of inhibitory interneuron function, including *DLX1*, *GAD1* and the *SLC32A1* vesicular GABA transporter, several Neurexin genes (NRXN) as key regulators for neuronal connectivity, and NMDA receptor subunits and other genes associated with glutamatergic signaling (Figure 6a; Supplementary Table 18). As this epigenetically defined subtype of genes potentially have important roles in PFC, we hypothesize that these genes could stand in crucial topological positions in a network such that many different pathways pass through these genes. Indeed, broadest neuron-specific H3K4me3-domain-associated genes outperformed the remaining group of genes in all five network test statistics (Supplementary Table 17). For example, we computed node ‘stress', which measures how many shortest paths pass through a specific node. We found that these broadest neuron-specific H3K4me3 peak-associated genes have a median stress of 92 540, which is more than fours times higher than that of other genes in the network. We also computed node ‘betweenness', which essentially measures the proportion of shortest paths passing through a node when there are more than one shortest paths between two nodes. We found that neuron-specific genes have a median betweenness of 0.001136, which is six times larger than that of other genes (Supplementary Table 17). These results demonstrate that neuron-specific genes are involved in significantly more critical pathways than genes that are not neuron-specific in the PFC network (*P*<1e-3, Wilcoxon Rank-sum test). In addition, we compared the average shortest path length and the largest shortest path length (also known as ‘eccentricity') between the two groups of genes (Supplementary Table 17). The results revealed that broadest neuron-specific H3K4me3 peak-associated genes can reach all the other genes in the connected network in significantly smaller number of steps than other genes (*P*<1e-3, Wilcoxon Rank-sum test), suggesting that perturbations on these broadest peak-related genes could have stronger effects on the integrity of the network.^70^ Moreover, we found that neuron-specific genes have a significantly larger degree than other genes in the network (Supplementary Table 17). Finally, we constructed weighted gene co-expression network analyses^44, 71, 72^ on RNA-seq datasets from PFC of 173 subjects independent from the present study,^72^ to identify modules of highly co-expressed genes enriched with markers for neuronal cell type. We demonstrate that significant overlap between top 5% broadest H3K4me3 peaks in PFC neurons with gene co-expression networks related to synaptic transmission and neuronal activity (Figure 6b).

### Broadest PFC neuron peaks are sensitive to neurodevelopmental disease mechanisms

**Figure 7:**
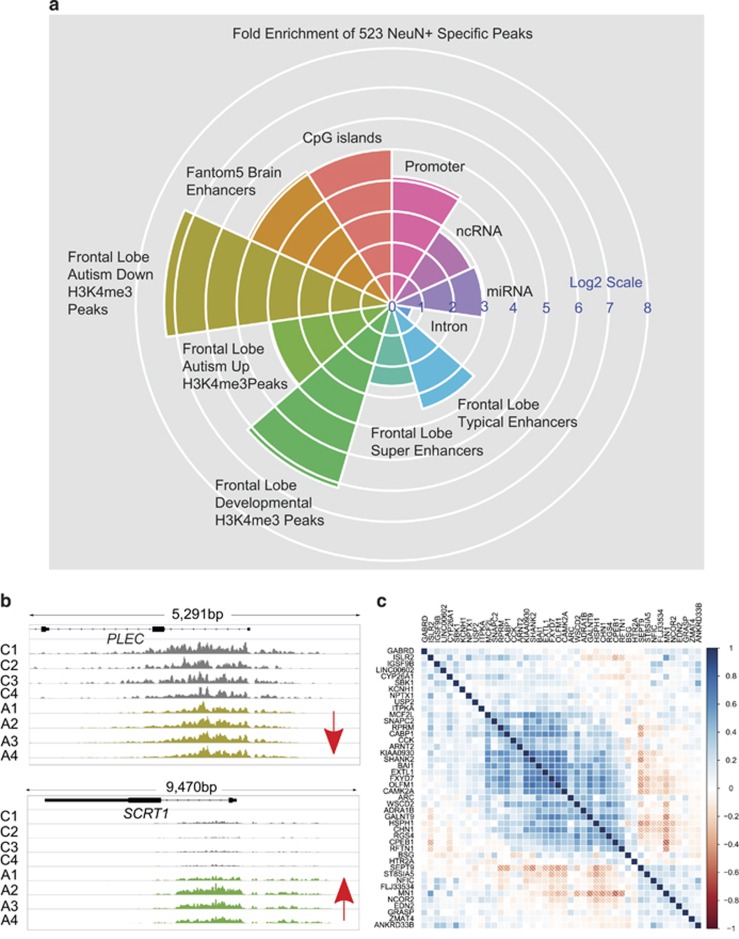
Broadest H3K4me3 peaks in neurons are developmentally regulated and enriched for super-enhancer and other *cis*-regulatory sequences. (**a**) Polar plot showing enrichment of top 5% broadest H3K4me3 peaks in PFC neurons for regulatory elements, including enhancers and super-enhancers,^14, 36, 37^ and published data sets on developmental and autism-associated H3K4me3 peak profiles in PFC neurons.^16, 18^ (**b**) Browser window showing in PFC neurons from controls C1–C4 and autism subjects A1–A4,^16^ representative examples for autism-associated broadest H3K4me3 peak, including *SCRT1* transcriptional repressor (and *PLEC* encoding a cytoskleleton-associated protein. SCRT1 up- and PLEC downregulated in PFC neurons of diseased subjects, as indicated. (**c**) Heatmap showing gene-to-gene correlations of subset of 44 transcripts associated with 45 top 5% broadest H3K4me3 peaks that show age-dependent regulation across the postnatal lifespan of adult human cerebral cortex (transcript levels from BrainSpan database^78^ and H3K4me3 peaks from Shulha *et al.*^18^). Notice large number of strong (dark blue) gene-to-gene correlations, suggesting that subset top 5% broadest H3K4me3-peak-associated transcripts are co-regulated from postnatal to old age. H3K4me3, H3-trimethyl lysine 4; miRNA, microRNA; ncRNA, noncoding RNA; PFC, prefrontal cortex.

Next, we employed the Genomic Association Test, a tool to assess overlap and enrichment between multiple sets of genomic intervals and includes corrections for gene density and chromosomal segments, among others.^35^ We wanted to examine the potential overlap of the broadest neuron-specific H3K4me3 peaks with functional elements including promoters and enhancers, and with previously published data sets on developmentally regulated H3K4me3-enriched loci in PFC neurons from controls^18^ and subjects on the autism spectrum.^16^ These data sets were particularly interesting from the viewpoint of the present study, given that deleterious mutations in regulators of H3K4 methylation rank prominently in exome-sequencing studies on neurodevelopmental disorders, including autism^15^ and schizophrenia.^73^ Indeed, there was a robust, 70- to 160-fold enrichment for sequences matching dynamically regulated H3K4me3 peaks, defined by extended age-related changes in PFC neurons, with inclining (or declining) levels of H3K4 methylation from the perinatal period to late childhood/early adulthood^18,16^ (Figure 7; Supplementary Tables 3A–D). Thus, 22/208 H3K4me3 peaks that were either missing or significantly decreased in a cohort of 16 autism cases (in comparison with control) were in the top 5% (broadest) peak category of PFC neurons (Supplementary Table 3B), in addition to 17/503 peaks abnormally increased in autism cases (Supplementary Table 3C). Furthermore, the top 5% broadest peaks showed a 17- to 34-fold enrichment for the promoter and brain-specific superenhancer sequences from the UCSC genome browser and FANTOM5 databases (Figure 7; Supplementary Tables 3E and F). Examples for disease-relevant broad H3K4me3 peaks include *DVL1* (Dishevelled Segment Polarity Protein 1), the neurotrophic peptide VGF and genes encoding ion channels and subunits such as *CACNA1C* and *GRIN2D*. To examine whether the striking developmental enrichment of the top 5% broadest H3K4me3 peaks was specific, we used to Genomic Association Test tool to conduct similar types of analyses for the top 5% tallest peaks. Strikingly, there was very little enrichment for developmentally regulated H3K4me3 peaks for the extremely tall peaks (Supplementary Table 19; Supplementary Figure 4), suggesting that such enrichment was highly specific for the top 5% broadest peaks in cortical neurons.

## Discussion

In the present study, we characterized the broadest H3K4me3 peaks from human PFC in the context of cell-type-specific regulation, association with neuronal and non-neuronal gene expression and potential implications for normal and diseased development. We first addressed the occurrence and the biological significance of the broadest H3K4me3 peaks in three different cell types, including NeuN+ PFC neurons, NeuN− PFC cells and nucleated blood cells. We identified novel regulators of these three different cell types by focusing on top 5% broadest H3K4me3 peaks (length in base pairs). Of note, the broadest H3K4me3 peaks, which in PFC neurons included >500 peaks in two different cohorts, showed a significantly stronger cell-type-specific signature compared with the complete pool of H3K4me3 peaks. Thus, broadest NeuN+ H3K4me3 peaks in the present study were enriched for genes regulating neuronal connectivity and signaling, including many ion channels, and synaptic plasticity and learning and memory. Broadest H3K4me3 peaks in non-neuronal PFCs showed enrichment for oligodendrocyte and other glial-related genes, in contrast to nucleated blood cells in which broadest peaks were associated with immune functions. The molecular regulators of the broadest H3K4me3 peaks remain to be determined for each cell type. Interestingly, we found that in PFC neurons, the DNA sequences of the top broadest 5% H3K4me3 peaks showed a significant enrichment for a set of motifs with binding affinity for several transcription factors and transcription factor families (Supplementary Table 8). These include, among others, transforming growth factor-β signaling-associated SMAD3, a nuclear protein associated with cell-type-specific master transcription factors^74^ and critically important for neuronal differentiation and morphogenesis,^75, 76^ and the *Meis1* homeobox transcription factor highly expressed in developing the forebrain including cortex.^77^ Interestingly, cross-species comparison of broadest H3K4me3 peaks in NeuN+ neurons of the adult cortex identified many genes regulating excitatory glutamatergic neurotransmission and dopaminergic pathways with a conserved broadest peak profile in human, non-human primates and mouse. It will be interesting to further explore in future studies the underlying mechanisms that resulted in this high degree of ‘epigenetic' conservation of glutamate- and dopamine-based signaling genes. For example, we would predict that regulatory sequences surrounding these genes are ‘exempt' from regulation by the H3K4-methyltransferase *Prdm9* and other molecules that remain functionally active in germline cells and are therefore considered ‘drivers' of mammalian speciation, including primates^65, 66^ and rodents.^67^ From a clinical perspective, the present study is in good agreement with genetic and postmortem brain studies implicating dysregulated H3K4 methylation to neurodevelopmental disease,^15, 16^ given that a significant portion of the top 5% H3K4me3 peaks in PFC neurons enriched in developmental data sets of H3K4me3 peaks. Therefore, at least some of the top 5% broadest H3K4me3 peaks appear to be sensitive to cellular mechanisms operating during an extended period of prefrontal development and maturation from birth to infancy to early and late childhood. Consistent with this hypothesis, the broadest domain H3K4me3 peaks are, according to our Bayesian network analysis, centrally located in a network of 7000 genes associated with PFC function in control (‘healthy' because non-demented) subjects. On the basis of the results of the present study, a more detailed analyses of specific histone modification profiles, including spread and breadth of histone H3K4 and other lysine methylation markings in specific cell types, bears promising potential to deliver valuable insights into epigenetic mechanism of normal and diseased brain development and aging. Such type of approaches, in the ‘Big data era' of functional genomics with NIH-sponsored consortia such as PsychENCODE consortium (http://www.psychencode.org) charting brain epigenomes and transcriptomes in hundreds of specimens across the lifespan, are likely to provide critical insights into the neurobiology of psychiatric disorders such as autism and schizophrenia.^79, 80, 81^
