# Supplementary material for: Deciphering H3K4me3 broad domains associated with gene-regulatory networks and conserved epigenomic landscapes in the human brain
Source: Transl Psychiatry. 2015 Nov 17;5(11):e679–. doi: 10.1038/tp.2015.169 (PMC5068762; doi:10.1038/tp.2015.169)
Supplement: Supplementary Information [file tp2015169x1.docx]

*Dincer et al.*

Deciphering H3K4me3 Broad Domains Associated With Gene Regulatory Networks and Conserved Epigenomic Landscapes in the Human Brain

**Inventory of Supplemental Tables and Figures**

**Supplemental Tables**

1. *Supplemental Table 1 : Overview on ChIP-seq and RNA-seq datasets*

**A**: Demographics and datasets on human prefrontal cortex, PFC for ChIP-seq.

**B**: Demographics and datasets on human blood for ChIP-seq.

**C**: Demographics and datasets on human prefrontal cortex for RNA-seq.

**D**: Datasets on animal cerebral cortex for ChIP-seq.

2.*Supplemental Table 2 : Alignment statistics for ChIP-seq and RNA-seq libraries*

**A**: human ChIP-seq libraries

**B**: human RNA-seq libraries

**C**: animal ChIP-seq libraries

3.*Supplemental Table 3 : Associations of top 5% broadest NeuN+ H3K4me3 peaks*

**A**: Association of NeuN+ 523 (top 5% broadest) H3K4me3 peaks with promoters, introns, CpG Islands, exons, intergenic and centromeric sequences, and brain genomic data.

**B**: Autism down peaks overlapping with top 5% broadest NeuN+ H3K4me3 peaks.

**C**: Autism up peaks overlapping with top 5% broadest NeuN+ H3K4me3 peaks.

**D**: Developmentally regulated peaks overlapping with top 5% broadest NeuN+ H3K4me3 peaks.

**E**: Brain enhancers (Fantom 5) overlapping with top 5% broadest NeuN+ H3K4me3 peaks.

**F**: Superenhancers overlapping with top 5% broadest NeuN+ H3K4me3 peaks.

4.*Supplemental Table 4 : Coordinates of top 5% broadest NeuN+ H3K4me3 peaks*

5.*Supplemental Table 5 : Coordinates of top 5% broadest NeuN- H3K4me3 peaks*

6.*Supplemental Table 6 : Coordinates of top 5% broadest H3K4me3 peaks in peripheral blood cells*

7.*Supplemental Table 7 : Novel transcripts associated with intergenic sequences overlapping with top 5% broadest NeuN+ H3K4me3 peaks*

*8. Supplemental Table 8: Top ranking motifs associated with broadest NeuN+ H3K4me3 peaks*

*9. Supplemental Table 9: Top 5% tallest NeuN+ H3K4me3 peaks* (**A**, cohort 1; **B**, cohort 2)

*10. Supplemental Table 10: Top 5% tallest NeuN+ H3K4me3 peaks reproducible in cohorts 1 and 2*

*11. Supplemental Table 11: DAVID pathway enrichment in top 5% tallest NeuN+ H3K4me3 peaks*

*12. Supplemental Table 12: GREAT functional annotation enrichments in among top 5% broadest H3K4me3* peaks (**A**, NeuN+; **B**, NeuN-; **C**, blood, **D**; common peaks across tissues).

*13. Supplemental Table 13: HG19 coordinates of top% 5 broadest NeuN+ H3K4me3 peaks across four mammalian species*

*14. Supplemental Table 14: Ingenuity pathway analysis of conserved broadest NeuN+ H3K4me3 peaks*

*15. Supplemental Table 15: Upstream Regulators of conserved broadest NeuN+ H3K4me3 peaks*

*16. Supplemental Table 16: Network analyses of conserved broadest NeuN+ H3K4me3 peaks*

*17. Supplemental Table 17: Network statistics for broadest NeuN+ H3K4me3 peaks*

*18. Supplemental Table 18: Prefrontal cortex Bayesian network statistics*

*19. Supplemental Table 19: Genomic Association Tester empirical p-values for top 5% broadest and top 5% tallest NeuN+ H3K4me3 peaks*

**Supplemental Figures**

1. *Supplemental Figure 1 : Features associated with top 1% broadest H3K4me3 peaks*

2*. Supplemental Figure 2 : Heatmap from mouse cortex transcriptome for transcripts associated with broadest H3K4me3 peaks*

1. *Supplemental Figure 3: Summary of average peak lengths of H3K4me3 NeuN+ peaks across species*
2. *Supplemental Figure 4: Enrichments for top 5% tallest NeuN+ H3K4me3 peaks*
