# Supplementary figures and images for: Deciphering H3K4me3 broad domains associated with gene-regulatory networks and conserved epigenomic landscapes in the human brain
Source: Transl Psychiatry. 2015 Nov 17;5(11):e679–. doi: 10.1038/tp.2015.169 (PMC5068762; doi:10.1038/tp.2015.169)

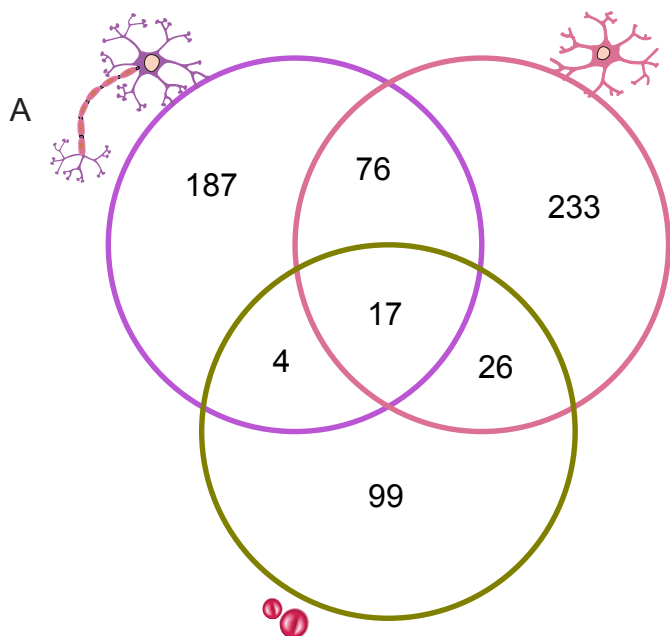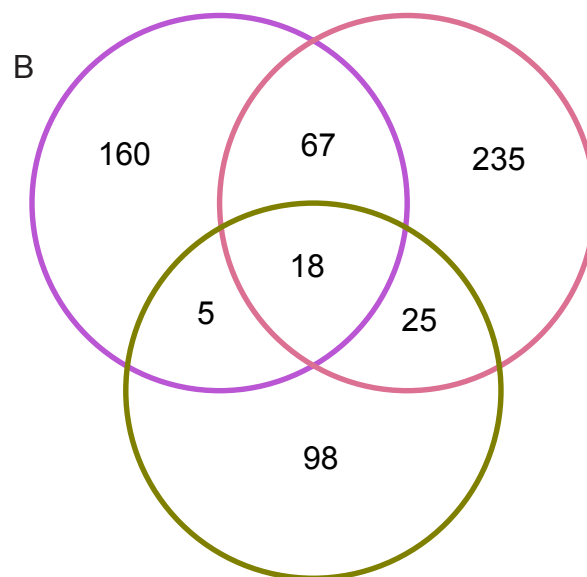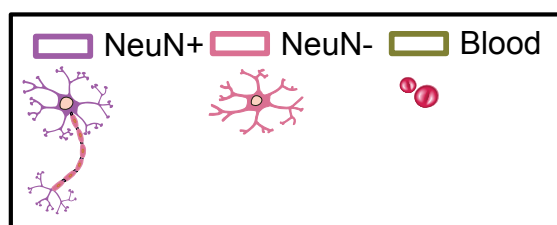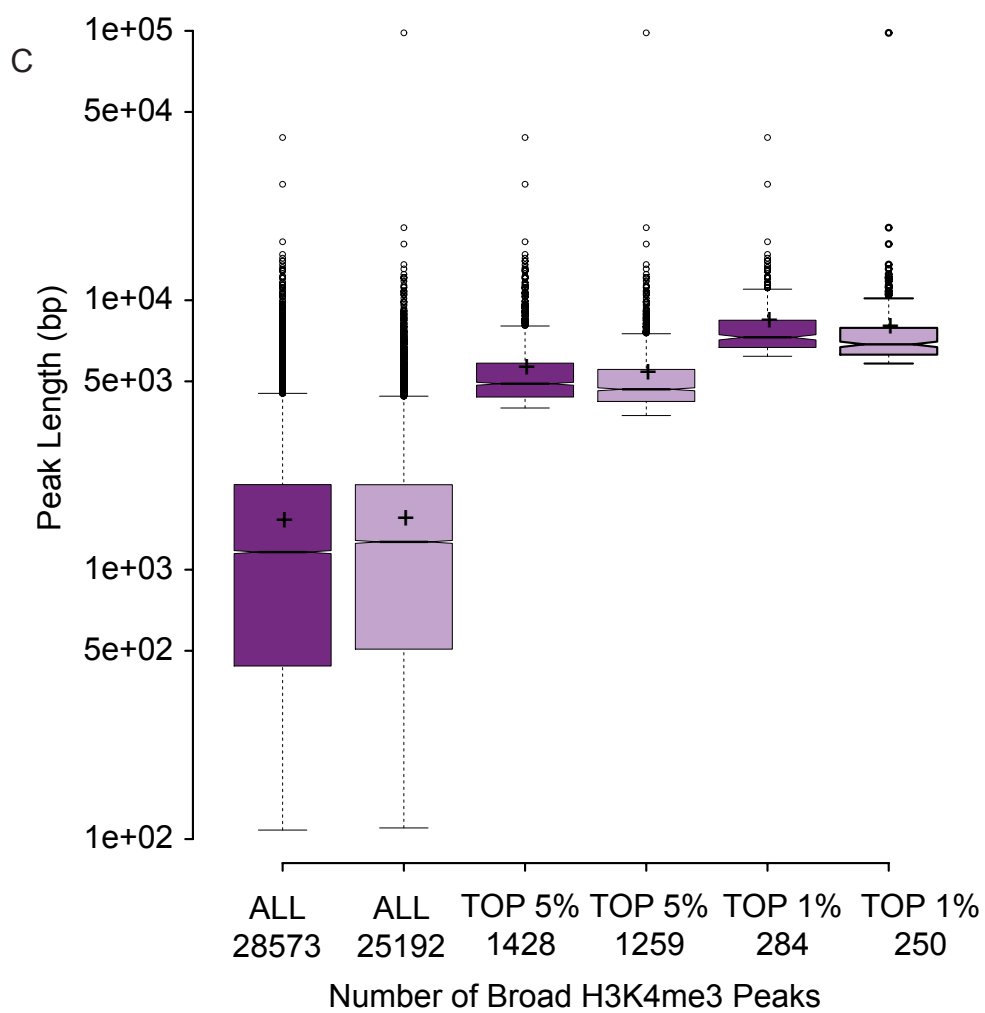

Supplement: Supplementary Figure 1 [file tp2015169x3.pdf]

Color Key

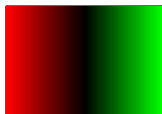

-4

0

4

Value

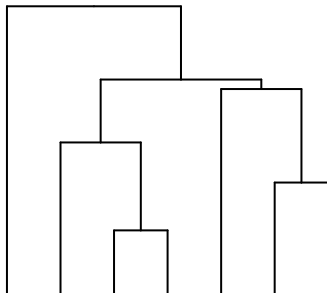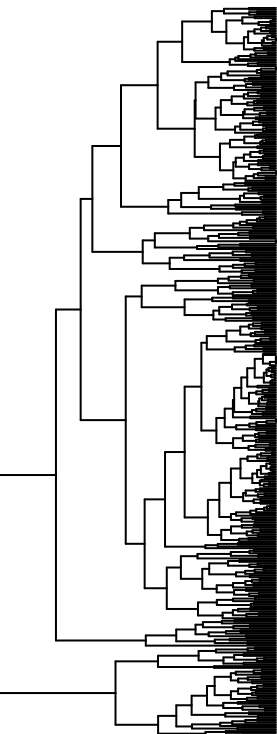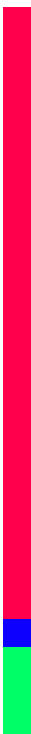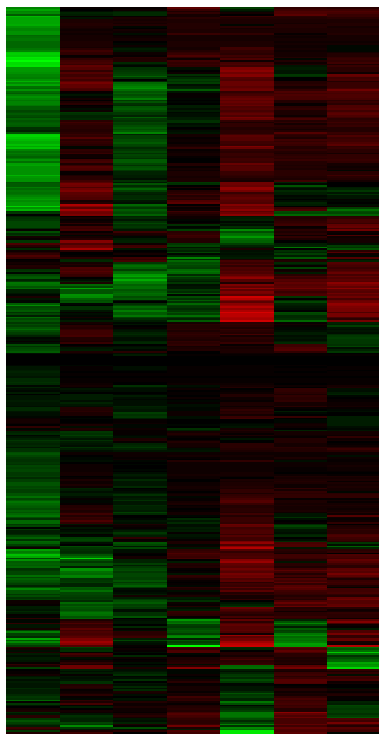

NEURON

ASTRO

OLIGO

NEWOLIGO

ENDO

MYELINOLIGO

MICROGLIA

Supplement: Supplementary Figure 2 [file tp2015169x4.pdf]

All Peaks vs Top 5% Peaks

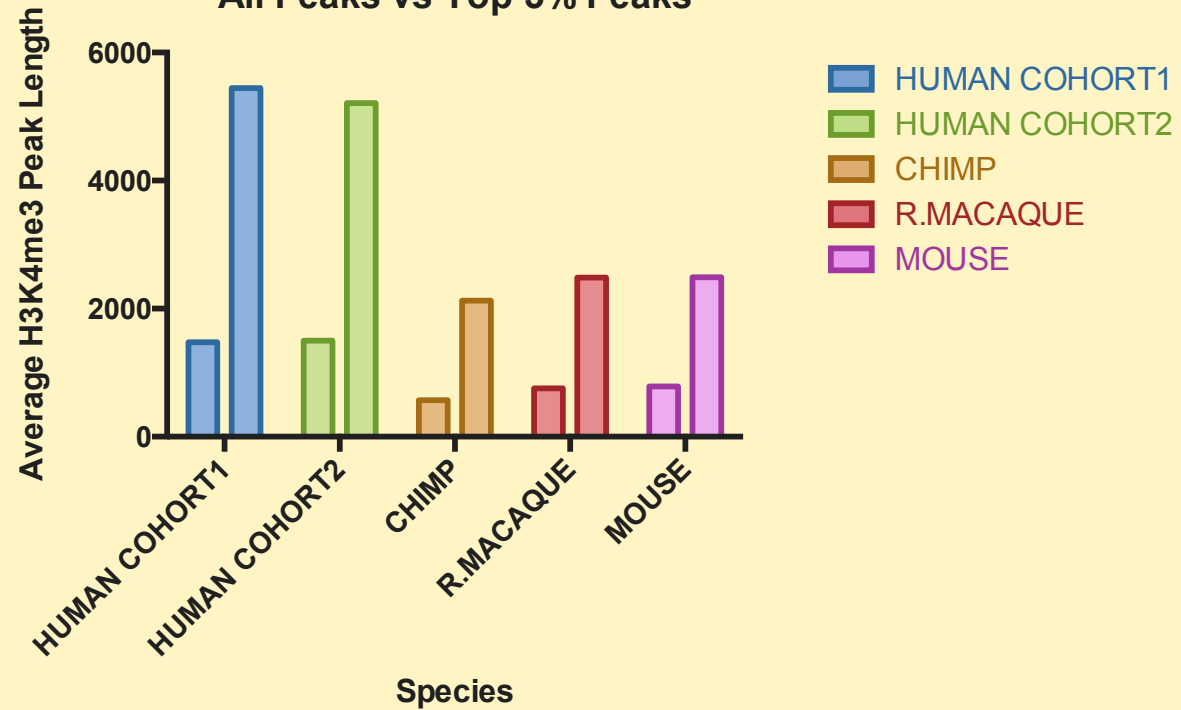

Supplement: Supplementary Figure 3 [file tp2015169x5.pdf]

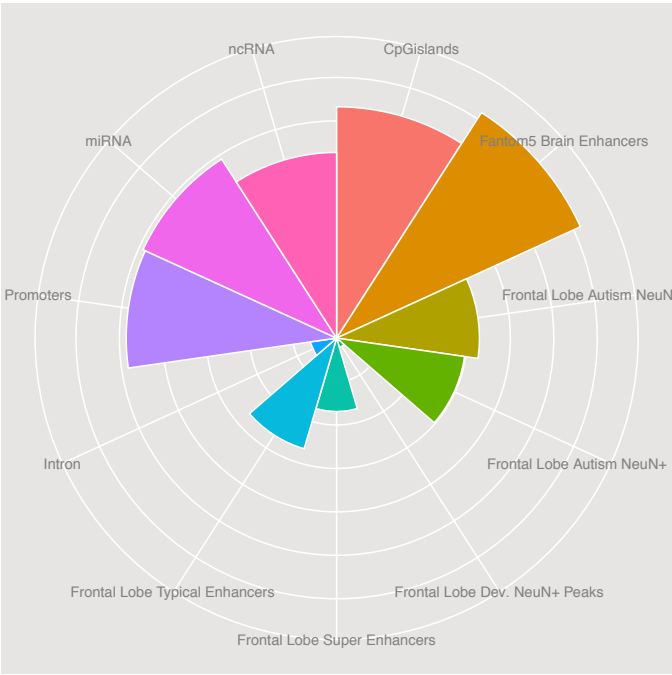

Supplement: Supplementary Figure 4 [file tp2015169x6.pdf]
